# Guanidine‐Based Ligands in Bioinorganic Chemistry: Coordination Modes and Applications in Anticancer Metallodrugs

**DOI:** 10.1155/bca/6568610

**Published:** 2026-04-19

**Authors:** Almudena del Campo-Balguerías, Alberto Ocaña, Iván Bravo, Carlos Alonso-Moreno

**Affiliations:** ^1^ Department of Inorganic, Organic and Biochemistry, Faculty of Pharmacy-Center for Innovation in Advanced Chemistry (ORFEO-CINQA), nanoDrug Unit, University of Castilla-La Mancha, Albacete, 02008, Spain, uclm.es; ^2^ Experimental Therapeutics in Cancer Unit, San Carlos Health Research Institute (IdISSC), Madrid, Spain; ^3^ Start Madrid–Jiménez Díaz Foundation (FJD) Early Phase Program, Jiménez Díaz Foundation Hospital, Madrid, Spain; ^4^ Department of Physical Chemistry, Faculty of Pharmacy, Nano Drug Unit, University of Castilla–La Mancha, Albacete, 02008, Spain

**Keywords:** antitumor agents, coordination chemistry, guanidine ligands, guanidines, medicinal inorganic chemistry, metallodrugs

## Abstract

Guanidine‐containing molecules represent a versatile class of nitrogen‐rich compounds whose unique structural and physicochemical features underpin a rich and tunable coordination chemistry. Their capacity to act as strong donor ligands, stabilize a variety of metal centers in different oxidation states, and access multiple coordination modes has established guanidine and guanidine‐like cores as pivotal components in medicinal inorganic chemistry. This review provides a focused overview of metallodrugs for antitumor applications in which guanidine or guanidine‐like ligands play a central role in metal coordination, highlighting how the coordination modes of the guanidine core translate into their application as anticancer metallodrugs. By correlating coordination mode, metal center, and ligand design with anticancer performance, this work underscores the potential of guanidine‐based ligand platforms for the development of next‐generation metal‐based therapeutics.

## 1. Introduction

**SCHEME 1:**
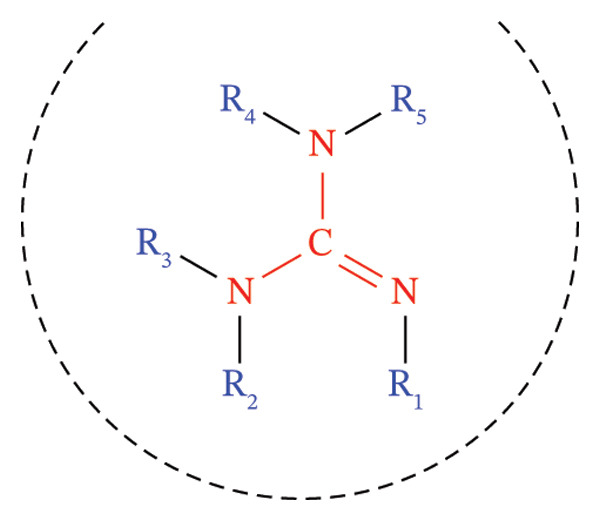
A general formula of the guanidine structure.

Guanidines are​ nitrogen‐rich organic molecules that play a significant role in various natural processes. Owing to their presence in numerous essential biological compounds, these molecules have remained a focal point of chemical research for more than 150 years following their discovery. The unique physical and chemical properties of guanidines stem from their structural configuration, which can be represented by the formula: R_1_–N=C(NR_2_R_3_) (NR_4_R_5_) (Scheme 1). The Y‐shaped CN_3_ configuration facilitates the straightforward formation of cationic derivatives upon protonation, yielding a guanidinium cation through resonance stabilization of the guanidine core. The guanidine core defines many compounds’ chemical and physicochemical properties, most of which are used in the health field.

According to their structure, guanidines can act as organic superbases [1], comparable to inorganic alkalis like sodium hydroxide [2]. Their chemical properties enable them to stabilize conjugated acids through resonance, making them an excellent and stable choice for this process [3]. The presence of both amine and imine functional groups allows these molecules to exhibit two distinct chemical properties: nucleophilic and electrophilic [2]. In this context, the guanidine moiety plays a crucial role in catalytic systems due to its ability to act as an intermediate for specific noncovalent interactions during ground‐state recognition events and to bind at the transition state in various catalytic processes [4]. The catalytic utility of guanidines in organic synthesis has been the subject of study by many researchers over the last few years. While much of the research has emphasized their synthetic applications, other studies have delved into the practical uses of bicyclic guanidine derivatives and investigated their nucleophilic properties as organocatalysts [5]. A key feature of these molecules derived from their characteristic structure is the high versatility of the guanidine scaffold, which can be readily and extensively modified. For example, chiral centers can be introduced at each of the three nitrogen atoms. Modifying the substituents during the synthetic process is a relatively straightforward strategy that enables fine‐tuning of both the electronic and steric properties [1]. Another notable feature of the guanidine moiety is the presence of nitrogen lone pairs, which enables its participation in the formation of metal complexes and strong hydrogen bonds, as well as its interaction with various electron‐deficient molecules [6–8]. The combination of these properties makes guanidines a compelling target for further exploration in the chemical sciences, with promising potential for applications in health‐related fields.

Beyond their synthetic and catalytic relevance, guanidine cores also occur broadly in nature, forming key structural motifs in amino acids, neurotransmitters, and diverse marine and terrestrial metabolites. Their capacity to engage in strong electrostatic and hydrogen‐bonding interactions underlies many of their biological roles and helps explain their evolutionary conservation [9, 10]. The historical development of guanidine chemistry, from early discoveries of creatine and guanine in the 19th century to the emergence of synthetic guanidine derivatives, ultimately laid the groundwork for a wide range of clinically relevant guanidine‐containing drugs [11, 12].

In parallel with the development of organic guanidine derivatives, medicinal inorganic chemistry has emerged as a rapidly evolving field within medicinal chemistry. This discipline focuses on the design, synthesis, characterization, and biological evaluation of inorganic metal‐based compounds, commonly referred to as metallodrugs, for therapeutic and diagnostic applications. The use of metal‐based compounds in biological systems exploits their distinctive chemical properties to address a wide range of diseases, particularly cancer. The versatility of metal centers allows them to adopt diverse coordination geometries, enabling specific and robust interactions with various biological targets. Furthermore, the redox properties of certain metal ions can be precisely modulated through the use of appropriate auxiliary ligands, facilitating tailored molecular interactions and diverse mechanisms of action. These distinctive features provide precise control over the physicochemical and stereochemical properties of metallodrugs, making them valuable tools in modern drug development [13–15].

Within this context, guanidine‐based ligands emerge as particularly promising scaffolds for the construction of metallodrugs. Their strong donor ability, structural versatility, tunable steric and electronic properties, and intrinsic biological activity make them attractive candidates for coordinating metal centers and modulating their pharmacological behavior. The capacity of the guanidine moiety to form stable metal complexes, combined with its well‐established synthetic accessibility, positions it at the interface between organic synthesis and medicinal inorganic chemistry.

This review first describes the main synthetic methodologies for the preparation of guanidine‐core structures, followed by an overview of research into the pharmaceutical applications of guanidine ligands in metallodrug development, with a particular focus on anticancer strategies.

## 2. Synthetic Methodologies for Guanidine‐Core Structures

The chemical synthesis of guanidines has been extensively studied and reviewed [16]. Synthetic methodologies can be broadly classified into two main strategies. The first involves classical synthesis, which relies on well‐established stoichiometric reactions using guanidinylating agents [16]. The second strategy encompasses catalytic metal‐mediated processes designed for the preparation of substituted guanidines.

### 2.1. Classical Synthesis

The traditional approach for synthesizing guanidines involves the chemical transformation of specific reagents using guanidinylating agents. This process typically entails the reaction of an amine with activated guanidine precursor groups, followed by deprotection steps to produce the final product.

Most notable examples of guanidinylating agents include thioureas, isothioureas, amide sulfonic acids, cyanamides, carbodiimides, triflyl guanidines, carboximidamide derivatives, and benzotriazoles [2, 16]. Among these, derivatives such as pyrazole‐1‐carboximidamide, S‐alkylisothiourea, and protected thiourea compounds are particularly common. The synthesis of di‐, tri‐ and tetra‐substituted guanidines is most simply achieved through the use of thioureas [17]. Isothioureas have been widely employed in the synthesis of guanidines in good yields, given their commercial availability and ease of preparation. Carbodiimides represent an intermediate stage in the synthesis of guanidines from thioureas. However, they can also be employed as reagents to facilitate the synthesis of the guanidine core. Despite being regarded as one of the most atom‐economical procedures, the restricted scope for generating a diverse range of guanidines, due to the requirement for aromatic carbodiimides and electron‐rich moieties, renders this classical method less attractive than others [16]. The main advantage of using pyrazole derivatives in these reactions lies in the fact that no toxic metals are required. However, the presence of protecting groups and electron‐withdrawing groups in the pyrazole ring is essential for the reaction to proceed. Despite this benefit, the method is not widely adopted due to several limitations, including the need for multiple reaction steps, low yields, and the high cost of the required reagents [16]. Additional methodologies are being studied for synthesizing aryl guanidines [18]. The use of cyanamide as a guanidinylating agent represents the most common approach; however, this method is often limited by the requirement of scandium (III) triflate in water and the toxicity associated with cyanogen bromide. Subsequent research for this purpose has focused on the use of benzotriazoles as efficient leaving groups to provide tetra‐substituted guanidines [19, 20].

### 2.2. Catalytic Metal‐Mediated Synthesis

**FIGURE 1:**
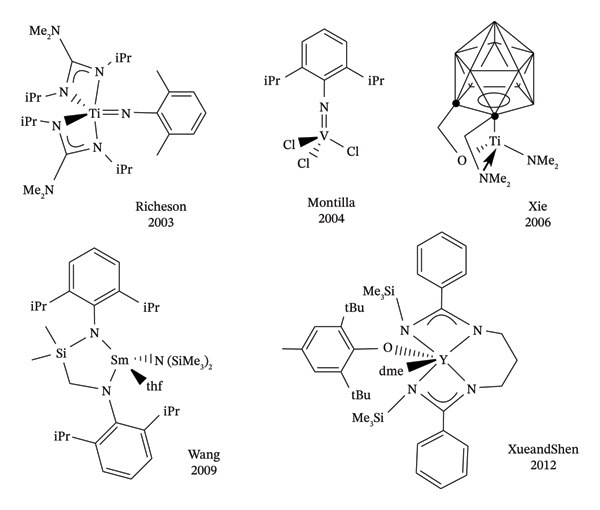
Representative chemical structures for successful guanidine catalysts.

The synthesis of N‐substituted guanidines via the reaction between carbodiimides and amines is widely regarded as one of the most efficient and straightforward methods. However, this approach is not universally applicable, as the reaction success depends severely on the nucleophilicity of the amines involved. Amines with insufficient nucleophilicity may fail to react, rendering this method ineffective. This limitation is particularly evident for aromatic amines, whose inherently low nucleophilicity prevents the reaction. To address these challenges, various catalytic strategies have been developed to enable the guanidinylation of different types of amines with carbodiimides [16]. The first catalytic method was reported in 2003 by Richeson et al. employing transition metal complexes as catalysts (see Figure 1 for representative successful catalyst structures) [21]. A key example is the catalytic guanidinylation process using imido titanium and vanadium complexes as the one reported by Montilla in 2004 (Figure 1), which enabled the insertion of amido‐metal derivatives into carbodiimides, effectively replacing the imido bond. This method facilitated the guanylation of both primary and secondary amines [22, 23].

Notably, the first atom‐economical route to substituted guanidines from less reactive aliphatic secondary amines was achieved using half‐sandwich alkyl complexes as catalysts [24]. These catalysts produced a diverse array of guanidines with functional groups such as C≡N, C≡CH, and aromatic C‐halogen bonds. Of particular interest, the half‐sandwich amido‐titanacarborane complex reported in 2006 by Xie (Figure 1) efficiently transformed heterocyclic, aliphatic, secondary acyclic, and cyclic amines into the corresponding guanidines [24]. Lanthanide complexes have also been extensively explored for guanidine synthesis, with alkyl and amide derivatives being especially significant. Among these, cyclopentadienyl‐free lanthanide amides, such as those developed by Wang (2009) and Xue and Shen (2012) (Figure 1), stand out due to their simplicity, availability, and exceptional catalytic efficiency. Operating under mild conditions with low catalyst loading, these complexes are highly effective, even for bulky aromatic and aliphatic secondary amines [25–27].

Recent advancements in the search for guanidinylating catalysts have focused on developing simpler and more versatile systems. Emerging options, such as ZnEt_2_, BuLi, [LiN(SiMe_3_)_2_], [AlCl_3_], [AlClMe_2_], and [Al(NMe_2_)_3_], have demonstrated high selectivity and efficiency in catalysis. These catalysts represent promising alternatives for synthesizing guanidines with diverse and broad applications [28–33].

## 3. Metallodrugs in Cancer: Design Principles and the Role of Ligands

As outlined in the Introduction, metal‐based compounds remain central to modern chemotherapy. Their structural diversity and tunable coordination chemistry enable mechanisms of action often inaccessible to purely organic drugs. In oncology, metallodrugs not only offer distinct therapeutic advantages but also present challenges related to toxicity, resistance, and selectivity. A clear understanding of their biological activity, activation pathways, and ligand design is therefore essential for the rational development of next‐generation metal‐based therapeutics.

In this context, coordinating ligands play a crucial role, as they strongly influence stability, reactivity, pharmacokinetics, and biological targeting. The following sections summarize key aspects of metallodrugs in cancer therapy, their mechanisms of action, and the strategic importance of ligand design, providing the foundation for the subsequent discussion of guanidine‐based systems.

### 3.1. Metallodrugs in Cancer Treatment

The therapeutic applications of metals can be traced back to the great civilizations of Egypt, China, Greece, and Rome. In the medical field, copper, gold, and silver were used as antiseptics, treatments for certain skin conditions and to promote wound healing and combat infection [13, 34]. Regarding cancer, metal‐based compounds have also played a longstanding role. Arsenic trioxide was recognized for its antileukemia activity. It was the mainstay of antileukemic treatment for much of the 18th and 19th centuries, until the early 20th century when radiotherapy and cytotoxic chemotherapy were introduced [35, 36]. However, it was not until the introduction of cisplatin (CDDP) for treating various types of cancer [37] that metal‐based compounds marked a significant breakthrough in oncology. CDDP was the first metal‐based anticancer agent introduced into clinical practice. This followed Barnett Rosenberg’s observation in the 1960s that platinum species released from electrodes inhibited the division of *Escherichia coli* and, later, tumor cells [13]. The drug was ultimately approved by the Food and Drug Administration (FDA) in 1978.

Nowadays, the most common therapeutic strategies currently in use for cancer treatment include surgery, radiation therapy, immunotherapy, and chemotherapy. Chemotherapy is the most widely applied approach. However, the effectiveness of this approach is often hindered by the development of drug resistance in cancer cells, which can result in treatment failure. Furthermore, antitumor medications are linked to notable side effects, including nephrotoxicity, neurotoxicity, and bone marrow suppression. Therefore, the development of novel pharmacological agents is of clinical importance to address these challenges and improve patient outcomes [38]. In the context of chemotherapy, CDDP, along with its second‐ and third‐generation derivatives carboplatin and oxaliplatin, continues to be extensively used to treat a wide range of cancers, including lung, colorectal, ovarian, testicular, bladder, and cervical cancers. However, their clinical use is often hindered by severe toxicities affecting the kidneys, nervous system, and auditory system, as well as the emergence of drug resistance during treatment [39]. Platinum‐based metallodrugs have played a foundational role, particularly with the development of Pt(IV) prodrugs that are reduced in vivo to active Pt(II) species. This prodrug strategy enhances plasma stability, improves tumor selectivity, and enables the incorporation of bioactive ligands that can further modulate therapeutic activity. The exploration of alternative transition‐metal complexes has expanded beyond platinum to include compounds based on ruthenium, palladium, copper, iron, rhenium, gold, and iridium [38].

### 3.2. Mechanisms of Action of Anticancer Metallodrugs

Despite the abundance of recently designed metal complexes for cancer treatment, the precise mechanisms of their antineoplastic activity remain largely unidentified. The limited understanding of these mechanisms is a key factor contributing to the slow progress in the clinical development and approval of metal‐based drugs. In contrast to conventional organic compounds, metal‐based agents often deviate from established drug‐like paradigms and display intricate thermodynamic and kinetic properties, which complicate their rational design and hinder their successful translation into clinical practice. Cationic metals are known to play a critical role in numerous essential biological functions and processes, as they can readily interact with electron‐rich biological molecules, such as proteins and DNA. The coordination of bioorganic compounds with metal cations has been shown to significantly alter the biochemical properties of both the metal cations and the ligand moieties. These moieties are known to exhibit a diverse range of bioactivities [40, 41].

Most metallodrugs used in cancer therapy are prodrugs that require activation inside cells. This activation typically occurs through hydrolysis, alterations in redox balance, or photoactivation. Hydrolysis is the most common mechanism in which water molecules replace labile ligands, such as chloride. For instance, the CDDP drug contains two chloride ligands that are relatively unstable. In the extracellular environment, the high chloride concentration stabilizes the compound and prevents hydrolysis. However, once inside the cell, the chloride concentration drops, allowing water molecules to replace the chloride ligands. The resulting aqua complex can interact with DNA, ultimately causing cell death [42]. Similar hydrolytic activation occurs in other metal complexes, including certain iridium(II), osmium(II), and ruthenium(II) derivatives. The rate of hydrolysis is influenced by the ligand type and is enhanced by chloride and electron‐donating groups [43]. Redox activation involves altering the cell’s redox balance, which is key to its survival, often by increasing reactive oxygen species (ROS) to induce cell death. Certain metallodrugs can influence the cellular redox state either directly, through redox reactions involving their metal centers or ligands, or indirectly, by interacting with biomolecules that regulate redox processes [44]. The low‐oxygen conditions typical of tumor tissues, along with the presence of biological reducing agents, such as GSH, NAD(P)H, or cysteine‐rich proteins, promote the reduction of metal complexes [45]. For instance, platinum(IV) prodrugs, including satraplatin and tetraplatin, are reduced in the hypoxic tumor environment to become active [46]. These prodrugs act by binding to DNA once reduced. Conversely, compounds containing ferrocene, such as ferroquine, promote ROS generation through oxidation via Fenton‐type reactions, which generate OH radicals [47]. The last method of activating metallodrugs is through photoactivation (phototherapy), which uses light to activate drugs. In cancer treatment, this encompasses photodynamic therapy (PDT), photothermal therapy (PTT), and photoactivated chemotherapy (PACT) [48].

### 3.3. Versatility of Metallodrugs and the Crucial Role of Ligands in Drug Design

The use of different metals, with their diverse oxidation states, coordination numbers, and geometries, has provided a broader range of tunable properties for metallodrugs compared to purely organic compounds. Beyond this inherent versatility, the ability to fine‐tune both the metal ion and its coordinating ligands significantly expands the potential for drug design, enabling the creation of a virtually limitless array of chemical structures with diverse biological activities. This structural diversity is driven by three closely interconnected factors: the choice of ligand, the nature of the metal center, and the oxidation state of the metal. Each of these elements plays a critical role in determining the physicochemical properties, pharmacokinetics, and biological activity of metal‐based drugs, both individually and in combination [49].

Ligands play a crucial role in modulating the reactivity, stability, and biological properties of metallodrugs. While the metal center and its oxidation state establish a fundamental framework for chemical behavior, it is the nature and spatial configuration of ligands, including their stereochemistry, that ultimately define the activity and specificity of the complex [49, 50]. A clear example of this is the contrast between CDDP and transplatin. While both complexes are square‐planar Pt(II) complexes, only CDDP, with its cis configuration of chloride and amine ligands, has been shown to have significant antitumor activity. Ligand lability is also a factor that impacts specificity. As demonstrated by the example of CDDP, the labile chloride ligands can be displaced not only by water but also by various biological molecules. This can reduce target specificity and increase side effects [51]. To address this, carboplatin incorporates a more stable bidentate carboxylate ligand, decreasing unintended interactions and prolonging circulation time [52]. In addition, the selection of ligands with hydrophilic functional groups has been shown to enhance the aqueous solubility of the metal complex. Conversely, to enhance the ability of a drug to cross the blood–brain barrier, a major challenge in treating brain tumors, more lipophilic ligands may be incorporated into the drug design [38]. Finally, noninnocent ligands, which are redox‐active, photoactive, or pharmacologically functional, can provide additional or controllable activity. In the context of PACT, for instance, a photolabile ligand can shield the metal center until exposure to light removes the protecting group, thereby enabling spatiotemporal control over the drug’s activation and therapeutic action [53].

The strategic selection of ligands, along with their modification through various substituents, has enabled the development of a broad spectrum of metal‐based compounds. Several of these have demonstrated high cytotoxicity while exhibiting improved pharmacokinetic properties [38].

### 3.4. Guanidine‐Core Ligands for the Synthesis of Metallodrugs as Antitumor Agents

Guanidine‐core ligands are highly attractive scaffolds for coordinating and developing various metal complexes due to their excellent donor properties. These ligands can stabilize a wide range of metals in different oxidation states, making them valuable components in bioinorganic chemistry [54, 55]. The characteristic Y‐shaped CN_3_ unit in the guanidine structure provides both steric and electronic flexibility, contributing to its versatility as a ligand. In this context, this review focuses on ligands that contain guanidine‐like functionalities as critical elements for metal coordination. Many ligands included in this review, although not necessarily containing a classical guanidine moiety, incorporate heterocyclic systems rich in nitrogen or other functionalities that mimic the electronic behavior and coordination capacity of guanidine. These guanidine‐like groups, whether embedded within heterocyclic frameworks, such as pteridines, triazolopyrimidines, or purine analogs, or present as exocyclic amino/imino functionalities, are central to their role as ligands. They act as the primary donor sites for metal coordination, often through nitrogen lone pairs, and contribute significantly to the stability, geometry, and electronic properties of the resulting metal complexes.

Guanidine‐based ligands exhibit a remarkably rich coordination chemistry that arises from the electronic delocalization within their characteristic Y‐shaped CN_3_ core. This delocalization endows guanidines and their deprotonated derivatives (guanidinates) with substantial σ‐donor strength and tunable steric flexibility, enabling them to bind metals with diverse structural outcomes [55].

Foundational coordination studies established that neutral guanidines lacking additional donor groups bind almost exclusively through the imino nitrogen, functioning as monodentate N‐donor ligands and preserving the planarity and resonance‐stabilized character of the CN_3_ core. This has been consistently demonstrated in classical systems such as Co(II), Cu(II), Zn(II), Pd(II), and Au(I) complexes, where coordination through N‐imine results in shortening of the C═N bond and partial equalization of the remaining C–N distances, confirming enhanced π‐delocalization upon metal binding [7]. The structural rigidity and electronic distribution within the guanidine unit, therefore, play a central role in dictating its coordination preferences and donor strength. When additional donor atoms, such as pyridyl, azo, phosphoryl, or nitrile groups, are incorporated into the ligand framework, guanidines can behave as bidentate or multidentate ligands, forming stable metallacycles or serving as bridging motifs between metal centers [7, 56].

A substantial shift in coordination behavior emerges upon deprotonation of the guanidine moiety. Monoanionic guanidinates, highly electron‐rich ligands, display markedly enhanced donor ability and adopt a wide diversity of binding modes, including monodentate κ^1^‐*N*, chelating κ^2^‐*N,N′*, and bridging μ‐*N*/μ‐*N,N′* motifs. Their electronic flexibility arises from resonance between 1,3‐diazaallyl and iminium/diamide forms, enabling the ligand to modulate its donor properties according to the requirements of the metal center. This capacity explains their compatibility with metals in high oxidation states and their ability to stabilize electron‐deficient species. Additionally, guanidinates support bridging modes (e.g., μ‐*N* or μ‐*N,N′*) in polynuclear complexes, which may enhance cooperative effects in targeting multisite biomolecules such as metalloenzymes or signaling proteins implicated in cancer proliferation [7, 55, 56].

Together, these findings confirm that guanidine‐based ligands, whether neutral, protonated, or anionic, form a continuum of coordination possibilities that can be strategically exploited in the design of metallodrugs. Their ability to stabilize diverse oxidation states, adopt multiple denticities, participate in intra‐ and intermolecular hydrogen‐bonding networks, and modulate metal‐centered electronics provides medicinal inorganic chemistry with a highly adaptable ligand platform for tuning key pharmacochemical parameters. In the context of metallodrug development, these coordination features directly influence molecular geometry, solubility, lipophilicity, redox behavior, and ligand‐exchange kinetics, and ultimately the biological activity and selectivity of metal complexes, reinforcing the importance of guanidine‐core ligands and guanidinates as versatile components for next‐generation anticancer agents and other therapeutic metal‐based frameworks.

In this work, guanidine‐based metallodrugs have been reviewed in which the guanidino group or the guanidine‐like groups mentioned above are central to their role as ligands for potential applications in cancer therapy.

#### 3.4.1. Monodentate Coordination of Guanidine‐Core Ligands

The vast majority of the reported metallodrugs fall into the category where the guanidine‐core molecule coordinates to the metal center through only one of its three nitrogen atoms [54, 57–209].

In most of the reviewed studies, which explore, among other aspects, the potential antitumor properties of these metal complexes, in vitro assays have been conducted to evaluate their effects on the viability of various cancer cell lines. In some cases, additional in vitro experiments, such as cell cycle arrest and apoptosis assays, have been performed to gain further insight into the mechanisms of action by which these metallodrugs exert their effects. However, only a small number of these studies progress to the in vivo stage. This discrepancy underscores the necessity for additional research in the field of medicinal inorganic chemistry, with a focus on identifying practical applications for newly synthesized metal complexes. In vivo studies represent the first major step required for any structure to be considered a viable candidate for antitumor therapy.

Regarding in vivo studies of novel platinum metallodrugs featuring a guanidine‐core structure coordinated to the metal in a monodentate fashion, Serebryanskaya et al. synthesized in 2013 twelve new chlorido guanidine‐core complexes of platinum(II) and palladium(II). The in vivo experiments of the lead compounds, conducted in mice bearing Ehrlich ascitic carcinoma, demonstrated more effectiveness than CDDP [119]. Hoffmann et al. in 2017 synthesized six novel guanidine‐core platinum(II) complexes. After extensive characterization and in vitro evaluations, in vivo testing showed less toxicity than CDDP or oxaliplatin [109].

Of the identified guanidine‐core ruthenium metallodrugs reported, five went on to perform in vivo studies. Velders et al. in 2004 synthesized and characterized three ruthenium(III) complexes of the NAMI‐A type. After detailed hydrolysis and in vitro evaluations, in vivo results showed that the lead compound significantly inhibited lung metastasis formation [152]. In 2018, Purushothaman et al. synthesized a novel guanidine‐core ruthenium(II) complex designed to target drug‐resistant cancer stem cells. The in vivo results demonstrated a significant tumor volume reduction of 55% and 80% in the low and high dose groups, respectively [161]. In 2020, Zhang et al. synthesized two ruthenium(II) complexes and formulated them into nanoparticles to improve stability. In vivo studies supported the strategy, yielding a successful outcome [164]. Recently, Chakraborty et al. synthesized eight novel guanidine‐based ruthenium(II) *p*‐cymene complexes. In vivo results demonstrated significant angiogenesis inhibition [169]. Finally, in 2024 the group of Chakraborty again synthesized twelve novel ruthenium(II) *p*‐cymene complexes based on five halogen‐substituted methyl and dimethylpyrazolyl‐benzimidazole ligands. The lead compound of the series showed potent NF‐κB inhibition and low in vivo toxicity, making it a promising candidate for therapeutic development [169]. These findings underscore the therapeutic potential of Ru(II) complexes as a low‐toxicity agent. They demonstrate the ability to overcome drug resistance, serve as a phototherapy agent, inhibit angiogenesis, and reduce tumor recurrence and metastasis.

Only one in vivo study has been found involving Zn complexes in which the metal is coordinated to the guanidine core in a monodentate fashion. In 2018, Vaden et al. synthesized a novel zinc‐binding small molecule, inspired by the natural marine product naamidine A. The compound acts as a zinc ionophore capable of selectively increasing intracellular Zn^2+^ concentrations in cancer cells while sparing untransformed cells. In vivo results demonstrated that this metallodrug, either as a standalone treatment or in combination with ZnSO_4_, significantly prolonged survival in mice bearing transplanted mammary tumors [179].

For the remaining metals, including copper, silver, iridium, palladium, gold, rhenium, iron, vanadium, chromium, cobalt, and nickel, no in vivo studies were identified. This indicates that the majority of research in this field has not yet progressed beyond in vitro evaluations.

#### 3.4.2. Chelating Coordination of Guanidine‐Core Ligands

**FIGURE 2:**
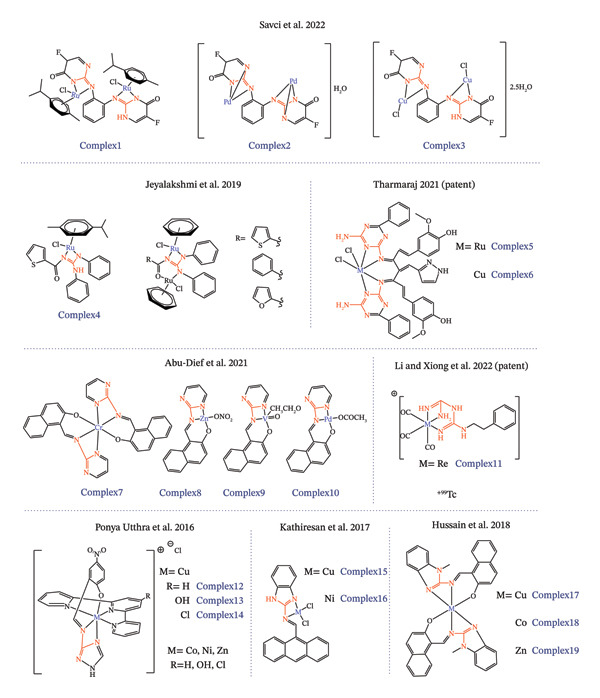
Structures of metallodrugs incorporating guanidine‐based chelating ligands for metal coordination in cancer therapy.

**FIGURE 3:**
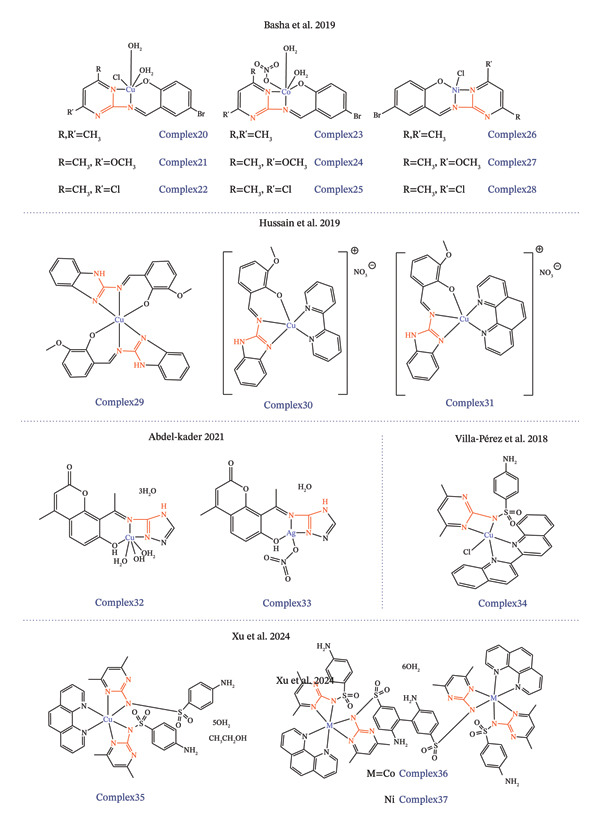
Continuation of structures of metallodrugs incorporating guanidine‐based chelating ligands for metal coordination in cancer therapy.

As discussed earlier in this work, guanidines and their anionic counterparts, guanidinates, serve as promising ligands for synthesizing metallodrugs. The monoanionic guanidinate form, often coordinating in an *N,N′*‐chelating mode, is particularly prevalent due to its electronic flexibility, which arises from extensive electron delocalization within the CN_3_ framework. Such stability is critical for metallodrug design, as it can improve resistance to ligand dissociation in physiological environments, enhance selectivity for biological targets, and minimize off‐target toxicity, thereby supporting their potential as effective antitumor agents. Only a few references have been identified concerning the bidentate, chelating coordination of guanidine‐core ligands to metals for the synthesis of antitumor metallodrugs (see Figures 2 and 3 and Table 1).

**TABLE 1:** κ^2^‐N,N‐Coordinated guanidine‐core metallodrugs with antitumor activity.

Authors	Cell lines tested	Complexes	IC_50_ (μM)	Outcomes	References
Savci et al. 2020	Caco‐2, L‐929 (nontumoral)	Complex 1 (Ru)	100.4	Complex 2 exhibits the highest antitumor potency, followed by complex 3. Complex 1 shows minimal cytotoxicity (IC_50_ ≈ 100 μM) Strong selectivity: Cu(II) over 9×; Pd(II) over 4.5× (lower toxicity to healthy cells) Electrochemotherapy (drug + electroporation) enhanced antitumor potency of both complex 2 and complex 3, outperforming 5‐FU	[141]
		Complex 2 (Pd)	25.35		
		Complex 3 (Cu)	31.88		

Jeyalakshmi et al. 2019	A549, HepG2, Vero (nontumoral)	Complex 4 (Ru)	176.3 (A549), > 250 (HepG2)	Complex 1 demonstrated only moderate cytotoxicity against A549 cells and was inactive against HepG2 and Vero cells. The remaining bimetallic Ru(II) complexes described in the study were found to be inactive across all tested cell lines Moderate DNA interaction of all four compounds Strong binding affinities of all complexes for BSA (bovine serum albumin) protein, with binding constants in the order of 10^4^ M^−1^	[215]

Tharmaraj P.M 2021 (patent)	A375, H357	Complex 5 (Ru)	—	89.25% cell death obtained at 250 μg/mL in H357 cell lines for complex 5 Complex 6 shows a close IC_50_ value compared to CDDP Through molecular docking with the EGFR‐TK receptor, complex 5 shows favorable binding (−421.56 kJ/mol)	[217]
		Complex 6 (Cu)	3.82 µg/mL (A375)		

Abu‐Dief et al. 2021	MCF‐7, HCT‐116, HepG‐2	Complex 7 (Cr)	8.45 (MCF‐7), 23.20 (HCT‐116), 19.25 (HepG‐2) (units in µg/mL)	Although less potent than vinblastine, complex 10 and complex 7 exhibited significant cytotoxicity DNA‐binding studies confirmed that all complexes interacted with CT‐DNA and complex 10 showed potential intercalation Molecular docking with 3HB5 revealed strong ionic and hydrogen‐bonding interactions, particularly for complex 9	[210]
		Complex 8 (Zn)	6.75 (MCF‐7), 17.85 (HCT‐116), 13.10 (HepG‐2) (units in μg/mL)		
		Complex 9 (V)	7.80 (MCF‐7), 20.20 (HCT‐116), 15.75 (HepG‐2) (units in μg/mL)		
		Complex 10 (Pd)	10.65 (MCF‐7), 25.10 (HCT‐116), 20.85 (HepG‐2) (units in μg/mL)		

Ponya Utthra et al. 2016	HeLa, Hep‐2, MCF‐7, NHDF (nontumoral)	Complex 12	0.87 (HeLa), 0.44 (Hep‐2), 0.68 (MCF‐7)	Complex 14 showed the highest cytotoxicity, particularly against Hep‐2, followed by HeLa and MCF‐7 All complexes exhibited comparable or better activity than CDDP in cancer cell lines, with lower toxicity to NHDF cells Complexes bind to CT‐DNA via intercalation, with complex 14 having the highest binding affinity, followed by complexes 13 and 12, all exceeding CDDP	[190]
		Complex 13	0.69 (HeLa), 0.56 (Hep‐2), 0.83 (MCF‐7)		
		Complex 14	0.48 (HeLa), 0.32 (Hep‐2), 0.91 (MCF‐7)		

Kathiresan et al. 2017	AGS	Complex 15 (Cu)	10.2	Complexes 15 and 16 interact with HS‐DNA via partial intercalation, with complex 15 showing higher binding affinity than 16 Both complexes induce apoptosis in AGS cells at 25 μM with complex 15 demonstrating a stronger apoptotic effect (more pronounced DNA fragmentation)	[214]
		Complex 16 (Ni)	23.6		

Hussain et al. 2018	HepG2, SK‐MEL‐1, HT018, HeLa, MDA‐MB231	Complex 17 (Cu)	26 (HepG2), 54.7 (SK‐MEL‐1), 29 (HT018), 23 (HeLa), 22 (MDA‐MB231)	Complex 19 triggered both early and late apoptosis, with the greatest efficacy observed in the MDA‐MB231 cell line Complex 19 inhibited adhesion and migration at IC_50_ concentrations with variable inhibition across cell lines In vivo studies Hematological toxicity: mild for complexes 17 and 18 and minimal for complex 19 (WBC/neutrophils spared in males) Hepatotoxicity: mild; ↑ SGOT, SGPT, GGT, ALP; and bilirubin normal Renal impact: low and slight ↑ urea only	[176]
		Complex 18 (Co)	45 (HepG2), 38 (SK‐MEL‐1), 51.3 (HT018), 44.5 (HeLa), 54.6 (MDA‐MB231)		
		Complex 19 (Zn)	12 (HepG2), 11.1 (SK‐MEL‐1), 15.8 (HT018), 8.3 (HeLa), 6.66 (MDA‐MB231)		

Basha et al. 2019	HepG‐2, HCT‐116	Complex 20 (Cu)	9.8 (HepG‐2), 11.2 (HCT‐116)	All complexes bind to CT‐DNA via intercalation with metal complexes exhibiting higher DNA binding affinity than free ligands Metal complexes show higher antitumor activity than free ligands (HepG‐2 and HCT‐116) with metal complexes potency order: Cu(II) > Co(II) > Ni(II) Strongest antitumor effect of CuL3 with IC_50_ of 7.46 μg/mL (HepG‐2), close to reference drug (vinblastine)	[219]
		Complex 21 (Cu)	8.5 (HepG‐2), 10.3 (HCT‐116)		
		Complex 22 (Cu)	7.46 (HepG‐2), 9.1 (HCT‐116)		
		Complex 23 (Co)	12.5 (HepG‐2), 15.8 (HCT‐116)		
		Complex 24 (Co)	11.7 (HepG‐2), 14.5 (HCT‐116)		
		Complex 25 (Co)	10.2 (HepG‐2), 12.9 (HCT‐116)		
		Complex 26 (Ni)	14.2 (HepG‐2), 17.3 (HCT‐116)		
		Complex 27 (Ni)	13.9 (HepG‐2), 16.8 (HCT‐116)		
		Complex 28 (Ni)	12.8 (HepG‐2), 15.4 (HCT‐116)		

Hussain et al. 2019	MCF‐7	Complex 29 (Cu)	≈100	Strong binding affinity of all complexes to HAS Apoptosis induced by ROS generation. GSH depletion confirmed as mechanism In vivo studies: complexes 29 and 31 display potent dose‐dependent anti‐inflammatory effects, unlike complex 30, which displays short anti‐inflammatory effect. Temperature decrease: complex 30 decreases temperature at lower doses (50 mg/kg) compared to complexes 29 and 31	[220]
		Complex 30 (Cu)	≈50		
		Complex 31 (Cu)	≈25		

S.Abdel‐Kader 2021	MCF‐7, HCT‐116	Complex 32 (Cu)	—	Complex 33 shows higher cytotoxic potency compared to its ligand (IC_50_ = 23.29 μM (MCF‐7 and HCT‐116) vs IC_50_ > 325.40 μM (MCF‐7 and HCT‐116, respectively) CDDP overcomes the cytotoxicity of complex 33 (IC_50_ = 5.64 μM MCF‐7, 17.73 μM HCT‐116)	[221]
		Complex 33 (Ag)	23.29 (MCF‐7), 23.29 (HCT‐116)		

Villa‐Pérez et al. 2018	MG‐63, A549	Complex 34 (Cu)	2.2 (MG‐63), 1.9 (A549)	IC_50_ of complex 34 is significantly lower than free ligands	[222]

Li and Xiong et al. 2022 (patent)	Mia Paca‐2	Complex 11 (Re)	39	Complex 11 exhibited up to 35‐fold greater cytotoxicity than phenformin It showed lower toxicity toward normal cell lines RAW264.7, NIH3T3, and hTERT‐HPNE In vivo studies: complex 11 demonstrated significant tumor size reduction, outperforming phenformin. No body weight loss or visible systemic toxicity was observed.	[218]

Xu et al. 2024	MDA‐MB‐231, SKOV3, HeLa, MCF‐7, A549, HepG2. HUVECs and LO2 nontumoral cells	Complex 35 (Cu)	2.62 (MDA‐MB‐231), 1.66 (SKOV3), 2.99 (HeLa), 1.98 (MCF‐7), 2.75 (A549), 2.67 (HepG2)	Complex 35 shows the highest inhibition of cell viability, superior to complex 36, complex 37, and *cis*‐dichlorodiammine platinum (CDDP) used as drug reference. It also demonstrates higher cellular uptake Complex 35 significantly induces apoptosis, increases ROS, and reduces MMP in MDA‐MB‐231 compared to complexes 36 and 37, which have weaker effects Complex 35 reduces VEGF/VEGFR2, NF‐κB, COX‐2, and inflammatory cytokines (IL‐1β and TNF‐α) In vivo studies: complex 35 inhibits TNBC tumor growth in mice and reduces microvessels (CD31) and inflammatory markers	[216]
		Complex 36 (Co)	27.50 (MDA‐MB‐231), 34.05 (SKOV3), 29.17 (HeLa), 17.15 (MCF‐7), 21.60 (A549), 21.47 (HepG2)		
		Complex 37 (Ni)	44.54 (MDA‐MB‐231), 37.69 (SKOV3), 25.24 (HeLa), 51.75 (MCF‐7), 44.70 (A549), 40.10 (HepG2)		

All identified metallodrugs featuring chelating guanidine‐core moieties have been assessed for their antitumor activity across various cancer cell lines. As shown in Table 1, no consistent pattern emerges in the selection of tumor cell lines tested in these studies, suggesting that research remains in a preliminary stage and that these complexes exhibit broad versatility rather than specific selectivity in their antitumor effects. Notably, colorectal, breast, and hepatocellular carcinoma cell lines appear more frequently than others. Among the studies reviewed, the work by Abu‐Dief et al. in 2021 stands out (Figure 2), evaluating the antitumor properties of zinc, vanadium, palladium, and chromium complexes against MCF‐7 (breast), HCT‐116 (colorectal), and HepG‐2 (hepatocellular carcinoma) cell lines [212]. The cytotoxicity of these complexes was then assessed against the three human cancer cell lines mentioned, using vinblastine as the reference drug. Cytotoxicity studies identified the palladium(II) complex as the most potent, whereas the zinc(II) and vanadium(IV) complexes were the least effective (see Table 1).

Another pattern to consider in the analysis of these studies is that more than half of them include DNA‐binding studies. Metal‐based drugs have long been recognized for their ability to target DNA, a central mechanism underlying their anticancer activity. Since the advent of CDDP, DNA has become one of the principal biological targets for antitumor agents, primarily through mechanisms involving DNA damage and crosslink formation. Likewise, compounds including palladium, ruthenium, and copper complexes interact with DNA through covalent (e.g., cross‐linking) or noncovalent (e.g., intercalation, groove binding, or electrostatic interactions) modes [213, 214]. Although the precise molecular interactions of many of these compounds have yet to be fully elucidated, their observed effects, such as inhibition of DNA replication, induction of SOS repair pathways, and suppression of RNA synthesis, strongly support their DNA‐targeting capabilities [214, 215]. Among the studies that evaluate DNA binding affinity is the work of Kathiresan et al. in which two novel mononuclear guanidine‐based Cu(II) and Ni(II) complexes were synthesized [210] (Figure 2). The complexes exhibited moderate to low cytotoxic potency compared to CDDP. Apoptosis assays demonstrated that both complexes induced cell death via apoptotic pathways in AGS cells, with complex 15 eliciting a more pronounced apoptotic response than complex 16. DNA‐binding studies suggest that, in addition to intercalative interactions, the complexes may also engage in covalent binding akin to CDDP, likely through substitution of labile chloride ligands by guanine N7 donor atoms [210]. Notably, a study developed in 2019 by Jeyalakshmi et al. synthesized a series of Ru(II)‐arene complexes incorporating N,N′,N″‐trisubstituted guanidine ligands, yielding monometallic and bimetallic ruthenium metallodrugs (Figure 2) [216]. DNA‐binding properties of the synthesized Ru(II)–arene complexes were investigated, demonstrating the ability to effectively bind to DNA, though with varying affinities [216].

Of the studies identified, only a few have progressed to in vivo evaluation, once again highlighting the significant gap between the design of metallodrugs and their transition to in vivo studies. The main outcomes of the studies are summarized in Table 1. Among the in vivo studies, it is worth noting a 2018 study conducted by Hussain et al. in which the safety profiles of three synthesized guanidine‐based Cu(II), Co(II), and Zn(II) metal complexes were evaluated through in vivo toxicity studies (Figure 3) [176]. The compounds exhibited some hematological and hepatic toxicity. However, minimal nephrotoxicity was observed. Furthermore, all three complexes caused increases in triglycerides, cholesterol, LDL, VLDL, and creatine kinase levels, suggesting changes in lipid profiles and mild cardiotoxicity. Among the three complexes, complex 19 displayed the lowest overall toxicity profile compared to classical platinum‐based drugs. Recently, Xu et al. evaluated the antitumor efficacy of a guanidine‐based copper(II) complex through in vivo experiments (Figure 3) [217]. The study compared the copper(II) complex with CDDP as the reference drug. After intraperitoneal injections, tumor volume was significantly reduced in the copper(II) complex group compared to the CDDP group. No significant organ toxicity was observed in major organs, with the copper(II) complex group showing less renal damage and splenic changes compared to the CDDP group.

Finally, as examples of the translation of guanidine‐based metallodrugs into clinical applications, two patents stand out. The first, from Thiagarajar College, was filed on February 3, 2021, and granted on February 23, 2024. Two transition metal complexes, Cu(II) and Ru(III), were synthesized, and their in vitro evaluation was conducted. Molecular docking revealed strong EGFR‐TK binding, supporting the anticancer potential of the complexes [218]. The second patent, from the University of Texas, was filed on March 9, 2022. The inventors synthesized biguanide complexes of rhenium and technetium‐99m. Specifically, the phenformin complexes Re‐Phen and ^99m^Tc‐Phen were evaluated for their antitumor properties, demonstrating up to 35‐fold greater cytotoxicity than phenformin in MiaPaca‐2 pancreatic cancer cells. In vivo studies assessing Re‐Phen efficacy showed significant tumor size reduction (greater than with *Phenformin*) with no significant weight loss or apparent toxicity observed. Additionally, ^99m^Tc‐Phen was evaluated for its use in SPECT/CT imaging [211].

## 4. Conclusions and Future Perspectives

The guanidine core is a highly versatile structural motif with distinctive physicochemical and biological properties that explain its long‐standing interest. Its resonance‐stabilized, nitrogen‐rich CN_3_ framework supports strong noncovalent interactions and dual nucleophilic/electrophilic behavior, making it common in many natural and synthetic compounds. These features, together with multiple nitrogen donors and electronic delocalization, also confer rich coordination behavior, allowing guanidines and guanidinates to function as strong, tunable ligands for many metal centers.

Within medicinal inorganic chemistry, these nitrogen‐rich molecules have emerged as valuable scaffolds for the rational design of antitumor metallodrugs. This chemical flexibility dictates their binding mode: neutral guanidines typically function as monodentate N‐donor ligands, while their deprotonated guanidinate counterparts commonly adopt κ^2^‐N,N′ chelating or bridging modes. Despite the continued clinical relevance of platinum‐based drugs, their use is significantly hampered by severe toxicities and acquired resistance, necessitating the urgent development of alternative metal‐based strategies. In this context, the guanidine framework offers an ideal platform for coordinating a variety of metals, stabilizing the metal center while enabling the precise fine‐tuning of the complex’s geometry and electronic properties. A growing body of preclinical studies has demonstrated that complexes of ruthenium, copper, zinc, and other metals supported by this ligand system exhibit promising antitumor activity coupled with potentially reduced toxicity compared to classical platinum agents. Though most efforts remain in early experimental stages, these results firmly establish the guanidine core as a highly attractive component for developing next‐generation metallodrugs capable of addressing the key limitations of conventional chemotherapy.

Despite this promise, several limitations and challenges are evident. The majority of guanidine‐based metallodrugs have been evaluated only in cell culture, with relatively few progressing to animal models, and mechanistic studies often stop at DNA binding, ROS generation, or generic apoptosis assays without fully elucidating molecular targets and pathways. In addition, the structural diversity of guanidine ligands and the heterogeneity of tested cell lines complicate the establishment of clear structure and activity relationships, underscoring the need for more systematic comparisons, standardized biological panels, and integrated mechanistic investigations. Future work should prioritize rational ligand design that explicitly links coordination mode and electronic structure to defined biological mechanisms, expand in vivo validation and toxicity profiling, and explore clinically relevant formulations.

Overall, guanidines and guanidinates stand out as highly adaptable coordination platforms for the development of next‐generation anticancer metallodrugs. Their continuum of coordination modes, capacity to stabilize reactive or high‐oxidation‐state metal centers, and ability to modulate key pharmacochemical parameters position guanidine‐based ligands as promising tools to address unmet needs in cancer therapy. Strengthening the bridge between coordination chemistry, medicinal inorganic design, and translational oncology will be essential to transform this rich chemical space into clinically relevant metal‐based therapeutics.

## Author Contributions

Almudena del Campo‐Balguerías: writing–original draft; Iván Bravo: supervision and conceptualization; Alberto Ocaña: writing–review and editing; Carlos Alonso‐Moreno: writing–review and editing, conceptualization, and funding acquisition.

## Funding

Iván Bravo and Carlos Alonso‐Moreno lab were supported by Grant 2025‐GRIN‐38324 funded by Universidad de Castilla‐La Mancha and ACEPAIN Foundation.

## Conflicts of Interest

The authors declare no conflicts of interest.

## Data Availability

All data supporting the results are included in the article.
